# Spatial heterogeneity and influencing factors of cognitive impairment among elderly hypertensive patients: evidence from rural communities in China

**DOI:** 10.1186/s12877-026-07587-4

**Published:** 2026-05-18

**Authors:** Jiaxin Han, Yudong Miao, Zhanlei Shen, Jingbao Zhang, Dongfang Zhu, Xinran Li, Mingyue Zhen, Jiajia Zhang, Jinxin Cui, Lingxiao Mou, Qingyong Lu, Yixi Wang, Jingwei Qin, Jingming Wei, Clifford Silver Tarimo, Qiuping Zhao, Rongmei Liu, Wenyong Dong

**Affiliations:** 1 Department of Health Management, College of Public Health, Zhengzhou University, Zhengzhou, 450001 Henan China; 2 Institute of Mental Health, Peking University Sixth Hospital, Beijing, 100191 China; 3 Henan Key Laboratory for Health Management of Chronic Diseases, Central China Fuwai Hospital, Central China Fuwai Hospital of Zhengzhou University, Zhengzhou, 451450 Henan China; 4 Henan Provincial People’s Hospital, Zhengzhou University People’s Hospital, Jinshui District, No. 7, Weiwu Road, Zhengzhou, 450003 Henan China

**Keywords:** Hypertension, Elderly, Cognitive impairment, Spatial heterogeneity

## Abstract

**Background:**

Cognitive impairment is frequent but often overlooked among elderly hypertensive individuals in rural settings. Existing studies have predominantly relied on global statistical models that assume uniform effects across space, failing to capture geographic heterogeneity in risk factor mechanisms and limiting the development of geographically targeted intervention strategies.

**Methods:**

A cross-sectional survey was conducted among 18,963 patients aged ≥ 65 years with diagnosed hypertension in Jia County, Henan Province, China (August 2023). The Bayesian Spatial Variable Coefficient (BSVC) model was applied to quantify each determinant’s spatial contribution using the Spatial Target Variable Contribution Index (STVPI). The Multi-Scale Geographically Weighted Regression (MGWR) model was subsequently employed to characterize the spatial scale and directional variation of each factor’s effect. Together, these complementary models enable both ranking of factor importance and mapping of spatially heterogeneous effects.

**Results:**

Central-northern rural communities exhibited the most pronounced high-high spatial clustering of cognitive impairment (Global Moran’s I = 0.17, *p* < 0.001). Lifestyle factors accounted for the largest share of spatial variation (25.34%), identifying behavioral determinants as the primary modifiable drivers of geographic disparities. Physical activity (STVPI = 8.62%, 95% CI: 5.64%–13.01%) and adequate sleep (STVPI = 5.21%, 95% CI: 2.26%–10.36%) demonstrated spatially stable protective effects consistent across all communities. In contrast, per capita household income, distance to major roads, and number of hospitalizations showed significant spatial heterogeneity, with effects varying substantially by location.

**Conclusion:**

Cognitive impairment among rural elderly hypertensive patients exhibits significant spatial clustering, with high-burden communities concentrated in the central-northern region. Physical activity and adequate sleep are spatially stable protective factors suitable for county-wide behavioral intervention, while income, healthcare access, and cooking environment require geographically differentiated responses—income support in north-central communities, inpatient care improvement in peripheral areas, and clean cooking promotion in western communities. The BSVC–MGWR framework provides a replicable tool for identifying high-risk areas and guiding precision resource allocation in resource-limited rural settings.

**Supplementary Information:**

The online version contains supplementary material available at 10.1186/s12877-026-07587-4.

## Introduction

Cognitive impairment is common among individuals with hypertension, with prevalence estimates ranging from 9.03% to 46.90% [1, 2]. This condition adds to the overall treatment burden, adversely affects disease prognosis, and may lead to a loss of functional independence and the emergence of behavioral challenges. Consequently, it significantly diminishes patients’ quality of life and autonomy, elevates the risk of hospitalization, and poses a serious threat to healthy life expectancy and overall well-being in the elderly demographic [3, 4]. In rural China, about 45% of elderly hypertensive patients experience varying degrees of cognitive impairment, affecting over 80 million individuals. This makes prevention and control of the condition an urgent public health priority [5, 6].

Research in health geography demonstrates that risk factors for chronic diseases vary across space, not only in their magnitude but also in their direction and underlying mechanisms [7]. Epidemiological and geospatial analyses conducted in rural regions of developing countries have further confirmed that chronic disease prevalence and associated risk factors exhibit significant spatial heterogeneity at the community level [8]. Existing evidence indicates a higher prevalence of cognitive impairment among hypertensive patients in northern rural China compared to urban populations, which is closely associated with lifestyle factors [9, 10]. This geographic variation suggests that spatial context—including socioeconomic conditions, environmental exposures, and access to healthcare—plays a fundamental role in shaping cognitive outcomes. Therefore, moving beyond global average estimates to characterize the spatial structure of cognitive impairment and its determinants is both theoretically important and practically necessary [11, 12].

Despite the significant health burden of cognitive impairment among elderly hypertensive patients in rural areas, three key gaps remain in the existing literature. First, current interventions rely heavily on pharmacological treatments and basic medical services, with limited integration of behavioral, environmental, and social structural factors, thereby constraining sustainable long-term outcomes [13]. Second, the development of non-pharmacological intervention strategies—such as lifestyle management and community environmental improvement—has been slow, and often lacks geographic and demographic specificity [14, 15]. Third, and most critically, existing research has predominantly estimated global average effects, systematically overlooking spatial heterogeneity in risk structures across different regions [16]. This omission has created practical challenges, including misaligned intervention targets and inefficient resource allocation in specific areas [17]. As a result, a critical theory-practice disconnect persists, limiting the effectiveness of cognitive impairment interventions.

The application of emerging spatial analytical methods provides new avenues for addressing these gaps [18, 19]. Although traditional Geographically Weighted Regression (GWR) can capture spatial non-stationarity, it assumes a single uniform bandwidth for all variables, preventing it from distinguishing the spatial scales at which different factors operate [20]. Multi-Scale Geographically Weighted Regression (MGWR) overcomes this limitation by assigning an optimal, variable-specific bandwidth to each predictor, thereby providing a more accurate representation of small-scale, community-level spatial variation [21]. Complementarily, the Bayesian Spatial Variable Coefficient (BSVC) model employs the Spatial Target Variable Contribution Index (STVPI) to quantify the spatial explanatory power of individual factors and identify core spatial influencers [22, 23]. While MGWR reveals where and at what scale factors matter, BSVC quantifies how much each factor contributes to overall spatial variation. The integration of both models thus enables a more comprehensive and policy-relevant spatial analysis.

This study utilizes Jia County in Henan Province—a pilot region for China’s grassroots healthcare reform—as a research setting. Jia County was selected because it serves as a national comprehensive pilot zone for primary healthcare, making it broadly representative of rural counties in central-northern China that share similar socioeconomic profiles, healthcare infrastructure, and demographic characteristics. While findings are specific to Jia County, the analytical framework and identified spatial patterns offer insights transferable to other rural settings in China and comparable low- and middle-income country contexts. Leveraging community survey data from a large-scale cohort of elderly hypertensive patients, we integrate BSVC and MGWR models to investigate the spatial distribution of cognitive impairment in rural areas and its underlying determinants. The research objectives are threefold: (1) to identify spatial high-risk clusters of cognitive impairment; (2) to reveal the spatial scales and differential contributions of key influencing factors; and (3) to provide methodological guidance and evidence to support the design of community-based intervention models tailored for rural older adults with hypertension and cognitive impairment. The findings are expected to provide theoretical basis and policy insights for the precise prevention and control of cognitive impairment in rural China and other low- and middle-income countries.

## Materials and methods

### Study region and data collection

#### Study region

**Fig. 1:**
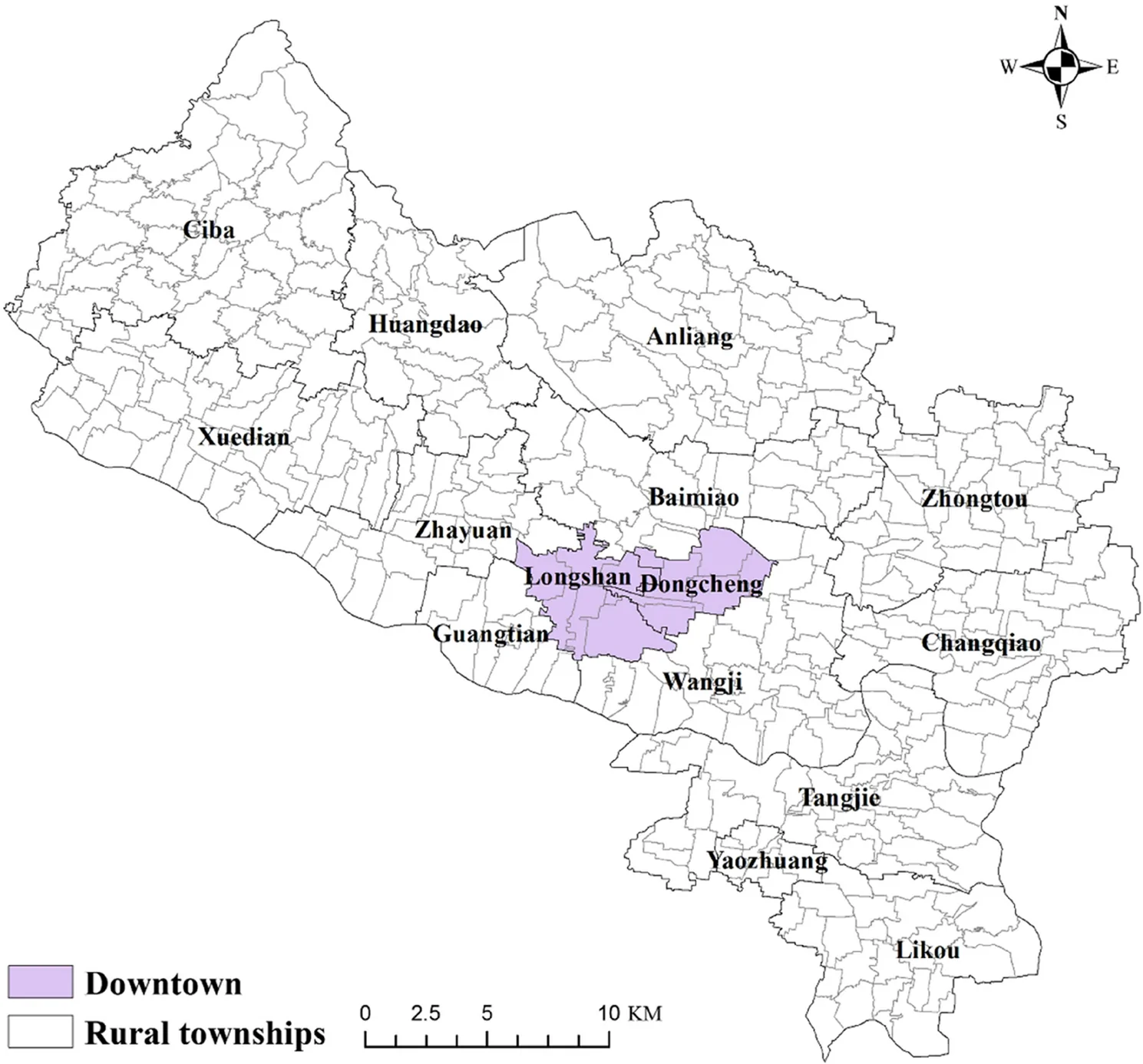
Administrative map of Jia County

The primary objective of this study was to investigate the prevalence of cognitive impairment and its associated risk factors among elderly hypertensive patients in rural China. The research was conducted in Jia County, which was purposively selected for three reasons: (1) its designation as a national comprehensive pilot zone for primary healthcare; (2) its demographic and socioeconomic profile representative of rural counties in central-northern Henan Province; and (3) the availability of complete community-level hypertension registries through the National Basic Public Health Database, enabling high spatial resolution analysis. Located in the central-western region of Henan Province, Jia County covers an area of 737 square kilometers and has a permanent population of 506,300. Its administrative structure comprises 2 sub-districts, 8 towns, and 5 townships. These include central urban areas (e.g., Longshan and Dongcheng) and rural townships (e.g., Ziba, Xuedian, Huangdao, Zhaoyuan, Guangtian, Anliang, Baimiao, Wangji, Zhongtou, Changqiao, Tangjie, Yaozhuang, and Lin Village), encompassing a total of 377 communities (urban census areas) and administrative villages (rural settlements) (see Fig. 1 and Supplementary Fig. 1).

#### Data collection

The study population, identified using cluster sampling, comprised all individuals aged 65 years and older with a diagnosis of hypertension recorded in the National Basic Public Health Database (NBPHD). The data were collected through a structured survey instrument that incorporated standardized, previously validated assessment scales and questionnaire modules derived from established national health surveys and clinical guidelines. From July to August 2023, a community-based cross-sectional survey was conducted in Jia County, Henan Province. The initial recruitment included 19,080 patients aged 65 years or older with a confirmed hypertension diagnosis. After excluding cases with missing information, 18,963 participants were retained for the final analysis. On-site, licensed physicians and trained research staff provided detailed explanations of the study to all participants, ascertained their current and historical use of antihypertensive medications, and requested to review all medications currently in use. Participants who were not adhering to a regular medication regimen or were taking medications contrary to prescription guidelines were excluded. The study cohort was comparable to the Seventh National Census data and the 2023 Chinese Geriatric Hypertension Management Guidelines in terms of hypertension prevalence and key socioeconomic characteristics, indicating strong sample representativeness (refer to Supplementary Table 1). The study protocol received approval from the Zhengzhou University Ethics Committee. All formally enrolled participants provided written informed consent and received a paper copy of the study protocol questionnaire prior to commencement.

#### Variable definitions

##### Outcome variable: cognitive impairment

The Montreal Cognitive Assessment (MoCA) was used to evaluate cognitive impairment [24]. According to previous studies, the MoCA test is more suitable than the Mini-Mental State Examination (MMSE) for detecting mild cognitive impairment in patients over 65 years old [25]. MoCA assesses eight domains: visuospatial/executive, naming, attention, language (language fluency), abstraction, delayed recall, and orientation. The test time is approximately controlled at 10–12 min. We used the widely used MoCA Beijing version with a total score of 30 and a cutoff of 26 for cognitive impairment. Participants scoring less than 26 were defined as cognitive impairment. For participants with less than 12 years of education, the total score was increased by one point.

##### Independent variable

Variable selection was informed by a comprehensive literature review and expert consultation, resulting in the inclusion of 20 indicators. These were organized into four primary categories: (1) sociodemographic characteristics (e.g., gender, education level, household income), (2) lifestyle behaviors (e.g., BMI, sleep patterns, physical activity), (3) healthcare utilization and history (e.g., annual hypertension-related expenditures, frequency of hospitalization), and (4) living environment and facilities (e.g., distance to major roads, access to clean sanitation and water). All continuous variables were Z-standardized prior to modeling. Categorical variables were dummy-coded as appropriate for the analytical requirements. The definitions and specific metrics for all independent variables are provided in Table 1.

**Table 1:** Socio-economic characteristics of the study participants (*N* = 18963)

Indicators (Unit)	Description of variables		Mean value			SD
cognitive impairment prevalence (%)	Proportion of community patients with current cognitive dysfunction		0.25			0.1
Population demographics						
Family sizes	The average number of people per household			4.69	3.76	
Per capita level of education (Year)	Years of schooling per capita			4.11	9.23	
Annual household income per capita (RMB yuan)	Total annual household income/Total household population			9002.98	5952.93	
Prevalence of household multimorbidity(%)	The presence of ≥ 2 chronic conditions among family members			0.19	0.08	
Lifestyle						
Obesity (%)	Number of obese patients (BMI ≧ 30)/Total number of patients			12.48	12.03	
Adequate sleep(%)	Percentage of patients in the community who slept 7–8 h per day			59.83	11.22	
Physical activity(%)	The proportion of patients who engaged in ≥ 150 min of moderate-intensity aerobic physical activity or ≥ 75 min of vigorous-intensity aerobic activity per week compared with all patients in the community			52.51	13.46	
Healthy diet(%)	According to the Healthy Eating Score adopted by the American Heart Association, meeting the following criteria was defined as: (1) total fruit and vegetable intake: ≥4.5 servings or servings per day; (2) fish intake ≥ 2 times per week; (3) red meat intake ≤ 5 times per week and processed meat intake ≤ 2 times per week Proportion of patients with a healthy diet compared with all patients in the community			55.27	11.15	
Smoking (%)	Proportion of current smokers compared with all patients in the community			24.58	8.58	
Drinking (%)	Proportion of current drinkers to all patients in the community			9.71	7.84	
Rate of blood pressure control(%)	Proportion of patients with controlled blood pressure			26.21	6.54	
Anxiety(%)	Proportion of community patients with GAD-7 score ≥ 10 (clinically significant anxiety symptoms)			0.37	0.12	
Health care						
Annual health expenditure per capita (RMB yuan)	Annual average health expenditure related to hypertension and its complications			2436.34	4836.14	
Disease duration(year)	Years since hypertension diagnosis			11.18	2.00	
Number of hospitalizations per capita	Per capita number of hospitalizations for hypertensive patients in the community			0.28	0.12	
WASH						
Access to clean cooking (%)	Percentage of patients in the community using clean energy sources such as natural gas for cooking			80.65	14.31	
Access to clean kitchen environment (%)	Percentage of community patients utilizing clean cooking equipment in household kitchens			81.25	15.21	
Access to safe water (%)	Percentage of patients in the community who receive safe drinking water from taps, water purifiers, etc. (treated and untreated)			67.87	25.89	
Access to sanitation facilities (%)	Percentage of community patients utilizing improved sanitation facilities			76.22	19.35	
Distance to major roads(m)	Residential proximity of hypertensive patients to primary traffic arteries			145.66	66.05	

Data are presented as mean (standard deviation) for continuous variables and frequency (percentage) for categorical variables. Per capita level of education and annual household income per capita exhibited skewed distributions (SD> Mean), which should be noted when interpreting these values. Mean and standard deviation are retained for consistency with community-level reporting conventions in comparable studies of rural Chinese populations

##### Definition of spatial units

For this study, administrative villages and communities served as the basic geographic units, from which 60 distinct spatial units were identified for analysis. The residential addresses of all survey participants were geocoded to the centroid of their respective village-level unit. Subsequently, a spatial connectivity matrix was constructed using the ArcGIS platform, followed by the generation of a spatial weights’ matrix for subsequent spatial regression modeling.

It should be noted that the use of administrative villages as spatial units raises potential concerns related to the Modifiable Areal Unit Problem (MAUP). Aggregating individual-level data to village-level units may mask within-village variation and introduce ecological bias. This aggregation level was chosen to align with China’s primary healthcare delivery system; future studies should conduct sensitivity analyses across alternative spatial scales to assess the robustness of observed spatial patterns.

### Methods

#### Spatial correlation analysis

Global spatial autocorrelation analysis was performed using GeoDa 1.20 software, primarily to examine global spatial autocorrelation (GSA) and Local Indicators of Spatial Association (LISA). GSA analysis characterizes the overall spatial distribution pattern across the study area, revealing the degree of dependency between attribute values and their spatial locations. A Moran’s I index greater than 0 indicates a positive spatial correlation, a value of 0 suggests a random spatial distribution, and a value less than 0 denotes a negative spatial correlation. The closer the absolute value of the index is to 1, the stronger the spatial autocorrelation and the more pronounced the clustering pattern. In this study, a Z-test was employed to evaluate spatial autocorrelation, with the significance level set at α = 0.05. A *p*-value of less than 0.05 was considered indicative of statistically significant global spatial autocorrelation. The specific formula is as follows:

1 $$I=\frac N{{\displaystyle\sum_i}{\displaystyle\sum_j}W_{ij}}\ast\frac{{\displaystyle\sum_{}}\left(X_i-\overline X\right)\left(X_j-\overline X\right)}{\displaystyle\underset{}{\sum_i\left(X_i-\overline{X}\right)^2}}$$

Where, $\:{X}_{i}$ and $\:{X}_{j}$ represent $\:{i}^{th}$ and $\:{j}^{th}$ community cognitive impairment prevalence, and $\:\stackrel{-}{X}$ is mean values of cognitive impairment prevalence across all communities. $\:{w}_{ij}$ represents the spatial weight or relationship between the $\:{i}^{th}$ and $\:{j}^{th}$ community. $\:{\sum\:}_{i}{\sum\:}_{j}{w}_{ij}$ represents the sum of all weights, and *N* represents the number of communities.

**Fig. 2:**
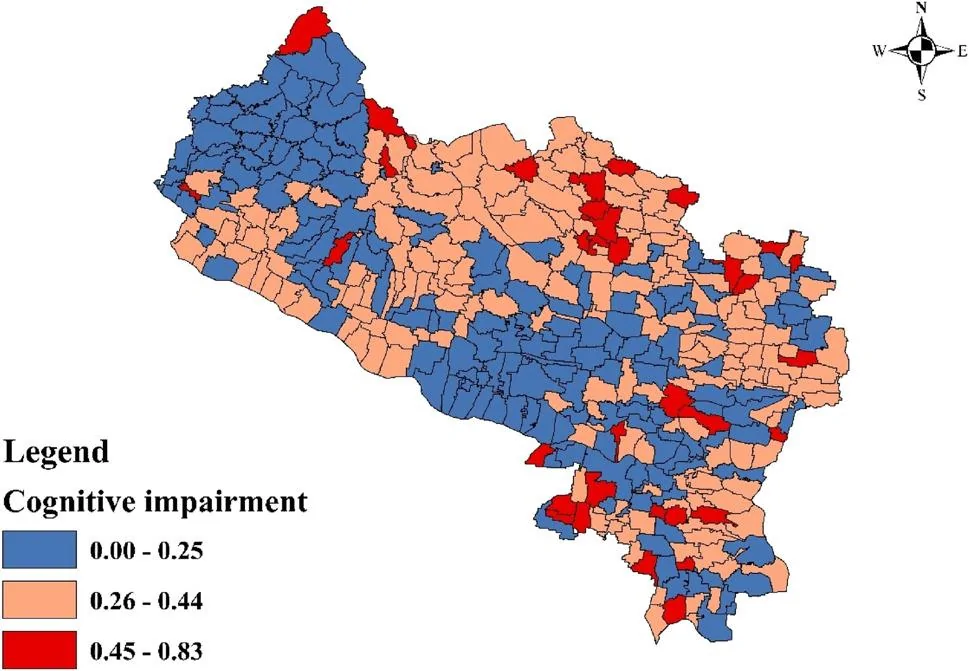
Spatial distribution of cognitive impairment prevalence among elderly hypertensive patients across village-level units in Jia County. Darker red shading indicates higher prevalence (range: 0.05–0.83); lighter blue shading indicates lower prevalence. The north-central region (Anliang Town) shows consistently elevated rates, while northwestern rural townships and central urban districts show consistently low rates

Using LISA analysis, the clusters were categorized into four types: “high-high,” “high-low,” “low-high,” and “low-low.” Clusters with positive spatial association (“high-high” and “low-low”) exhibit strong similarities with their neighbors, whereas those with negative spatial association (“high-low” and “low-high”) are identified as spatial outliers. Specifically, the “high-high” clusters signify areas where a community and its neighbors all exhibit higher-than-average rates of cognitive impairment in elderly hypertensive patients. Conversely, “low-low” clusters represent areas in which a community and its surrounding neighborhoods all demonstrate rates below the average. The “high-low” clusters denote communities with rates above the average that are surrounded by neighborhoods with rates below the average. Similarly, the “low-high” clusters describe communities with below-average rates that are adjacent to communities with above-average rates. It should be acknowledged that Moran’s I has inherent limitations: it treats communities near the mean similarly regardless of the absolute magnitude of deviation, and yields a value of zero when a community’s value exactly equals the spatial mean regardless of neighboring values. Therefore, LISA results should be treated as evidence of spatial non-randomness. Their interpretation should be made with reference to the continuous spatial distribution of raw prevalence (Fig. 2), avoiding absolutist conclusions drawn solely from cluster categories.

#### Linear regression model (OLS model)

The original data were processed using Z-score standardization to eliminate dimensional differences among variables. Subsequently, ordinary least squares (OLS) regression analysis was employed to identify key factors influencing cognitive impairment in elderly hypertensive patients.

#### Bayesian space variable coefficient model

BSVC model was employed to identify variables exhibiting spatial heterogeneity, specifically non-stationary spatial effects. The core of the BSVC model is the coefficient$\:{{\upbeta\:}}_{k}\left({s}_{i}\right)$, which denotes that regression coefficients are not globally fixed but vary across spatial locations$\:\left({s}_{i}\right)$ [26]. This formulation allows the model to capture geographic variations in the influence of explanatory variables on the dependent variable [23]. The statistical mechanism of the BSVC model relies on Bayesian methods to estimate these spatially varying coefficients, incorporating spatial smoothing priors (e.g., Gaussian processes) and a spatial weight matrix to introduce spatial autocorrelation.

The model was implemented using the R-INLA package (R version 4.3.1; INLA version 23.04.24) with weakly informative priors for fixed effects (Normal distribution, mean = 0, precision = 0.001) and penalized complexity (PC) priors for spatial random effects hyperparameters. Model convergence was assessed by examining effective sample sizes and potential scale reduction factors. Posterior marginal distributions were summarized using the median and 95% confidence interval (CI).

Key driving factors were identified by quantifying and comparing the interpretable percentages of different spatial heterogeneity factors, expressed as their spatial contribution or relative importance. Unlike mainstream methods that rank factors using absolute metrics, the STVPI serves as a relative evaluation metric, providing crucial prior evidence for spatial attribution. Variables with STVPI > 5% were identified as key contributing factors and subsequently entered into MGWR for spatial heterogeneity mapping. The formula is as follows:

2 $$\rho_k=\frac{\sigma_{\mu k}+\sigma_{\eta k}}{{\displaystyle\sum_{k=1}^K}\left(\sigma_{\mu k}+\sigma_{\eta k}\right)+\sigma_\in}\times100\%$$

where is $\:{\rho\:}_{k}\:$expressed as a percentage in the range of [0,100] for the *k*-th explanatory factor. $\:{{\upsigma\:}}_{{\upmu\:}k}$ represents the variance component of the spatially non-stationary random effect of the *k*-th explanatory factor at the standard deviation scale. The *k*-th explanatory factor’s temporally non-stationary random effect is represented by $\:{{\upsigma\:}}_{{\upeta\:}k}\:$at the standard deviation scale. $\:{{\upsigma\:}}_{\epsilon}\:$denotes the variance component of the unexplained random effect in the residual term. Uncertainty in STVPI estimates is quantified using 95% confidence interval derived from the posterior distribution of the variance components.

#### Multi-scale geographically weighted regression

Based on the BSVC-identified key contributing factors (STVPI > 5%), MGWR was subsequently applied to map the spatial distribution and heterogeneity of their effects across communities. MGWR extends traditional GWR by allowing each explanatory variable to be modeled at its own optimal spatial scale, rather than assuming a single uniform bandwidth for all variables. This is important because different factors may operate at different geographic scales: for example, lifestyle habits may reflect local community norms (narrow bandwidth), whereas economic influences may reflect broader regional patterns (wide bandwidth). By allowing variable-specific bandwidths, MGWR avoids scale misspecification inherent in standard GWR and provides more accurate characterization of spatial heterogeneity. MGWR was implemented using MGWR 2.2 software (pysal/mgwr, Python 3.8). The optimal bandwidth for each variable was selected by minimizing the corrected Akaike Information Criterion (AICc), and a nearest-neighbor-based bi-square kernel function was used to weight neighboring observations. Variables with large bandwidths (approaching the total sample size) and consistent coefficient signs across all spatial units were classified as spatially stable; those with small bandwidths and substantial coefficient variation were classified as spatially heterogeneous. Its mathematical expression is presented as follows.

3 $$Y_j=\beta_0\left(\mu_j,\nu_j\right)+\sum\limits_{i=1}^k\beta_i\left(\mu_j,\nu_j\right)x_{ij}+e_j$$

Where $\:{x}_{ij}$ is the local variable, $\:{e}_{j}\:$ is the random error term; $\:\left({u}_{i},{v}_{i}\right)$ is the spatial coordinate of the location of community * i*; $\:{\beta\:}_{j}$ is the estimation of the coefficient for community* i* and * j* is the optimal bandwidth size. Uncertainty in MGWR coefficient estimates is conveyed through the spatial distribution of local t-values and the interquartile range of local coefficients across communities.

The complementarity between BSVC and MGWR is as follows: BSVC quantifies how much each factor contributes to overall spatial variance (a global ranking of spatial importance), while MGWR maps where and at what scale each factor’s effect is strongest (a local characterization of spatial heterogeneity). Together, they provide both a summary ranking and a community-level spatial portrait of the risk landscape.

All data preprocessing and descriptive statistical analyses were conducted using Stata 17.0. Multicollinearity among variables was assessed using the variance inflation factor (VIF), and variables with a VIF greater than 10 were excluded. A two-sided *p*-value of less than 0.05 was considered statistically significant. Spatial visualizations and cartographic output were generated using ArcGIS 10.8.

## Results

### Descriptive statistics

The study included a total of 18,963 elderly hypertensive patients aged 65 years or older from rural areas. The mean age of participants was 71.4 years (SD = 5.9), and 57.7% were female. The overall prevalence of cognitive impairment was 25.42%, with substantial variation observed across communities. Among the participants, 1,913 (10.08%) resided in urban neighborhoods (Longshan and Dongcheng), while the majority (17,050, 89.92%) were distributed across rural towns and villages within Jia County (see Supplementary Fig. 2 and Supplementary Table 2).

### Spatial distribution of elderly patients with hypertension and cognitive impairment

As shown in Fig. 2, the prevalence of cognitive impairment exhibits distinct spatial clustering across village-level units. Low-rate clusters are primarily distributed in two types of areas: rural townships such as Ziba and Xuedian in the northwest of Jia County, and the central urban neighborhoods of Longshan and Dongcheng along with their surrounding suburban regions, forming contiguous low-prevalence zones. In contrast, areas with high rates are more scattered; notable concentrations are found in Anliang Town in the north-central region, where prevalence rates are consistently elevated, ranging from 0.45 to 0.83. Based on descriptive community-level data (Supplementary Table 2), this region is characterized by limited healthcare access, lower household income, and greater reliance on solid fuel cooking, which may collectively contribute to the elevated cognitive impairment burden observed there.

### Spatial autocorrelation of factors affecting cognitive impairment

**Fig. 3:**
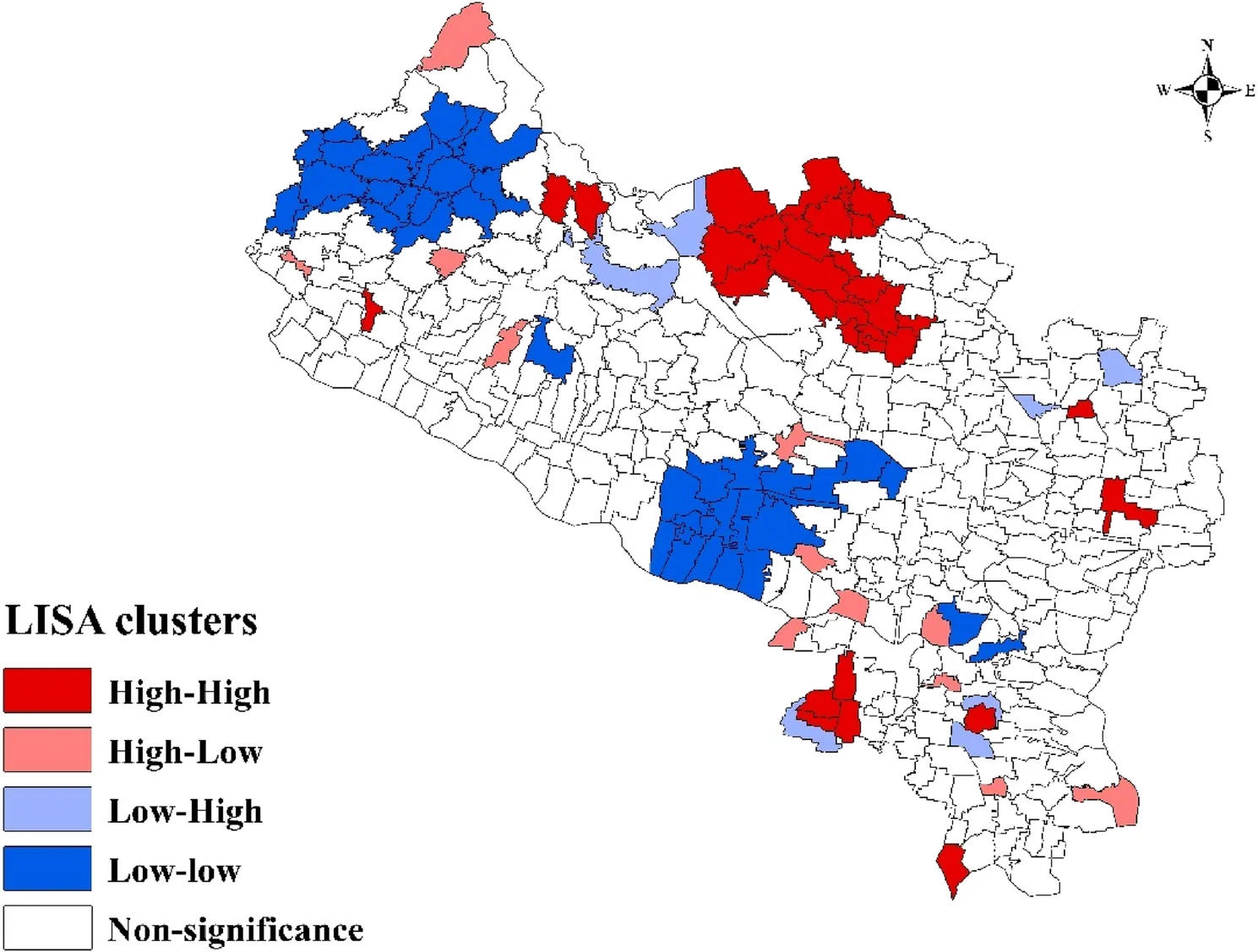
LISA cluster map of cognitive impairment rates among elderly hypertensive patients in Jia County. Red shading indicates high-high clusters (communities with high rates surrounded by high-rate neighbors); blue shading indicates low-low clusters (communities with low rates surrounded by low-rate neighbors); white indicates non-significant spatial association (*p* > 0.05). High-high clusters are concentrated in the north-central region (Anliang Town); low-low clusters are distributed in the northwest and central urban areas

Global spatial autocorrelation analysis yielded a Moran’s I of 0.17 (Z-score = 5.49, *p* < 0.001), confirming that the spatial distribution of cognitive impairment prevalence is significantly non-random and exhibits positive spatial autocorrelation. This means that geographically proximate communities tend to share similar cognitive impairment burden levels. The magnitude of Moran’s I (0.17), while moderate, is consistent with village-level health analyses in rural China where spatial clustering is constrained by within-county administrative boundaries. LISA analysis further revealed the geographic structure of this clustering (Fig. 3). High-high clusters were concentrated in rural communities in the central-northern region, particularly in and around Anliang Town, representing priority areas for targeted intervention. Low-low clusters were primarily identified in the northwestern rural communities (Ziba, Xuedian) and central urban districts (Longshan, Dongcheng). No significant high-low or low-high outlier clusters were identified, suggesting a relatively smooth spatial gradient across the county.

### Spatial contribution and spatial heterogeneity of influencing factors

#### BSVC model results: spatial contributions

The BSVC model explained 84.41% (95% CI: 79.35%–88.67%) of the spatial variation in cognitive impairment rates, with the remaining 15.59% attributable to residual unexplained variation. Among the four variable categories, lifestyle factors exhibited the greatest cumulative spatial contribution (25.34%), indicating that behavioral determinants are the dominant drivers of geographic disparities in cognitive impairment. This was followed by WASH factors (17.13%), sociodemographic factors (16.78%), and healthcare utilization factors (10.57%).Among individual variables, per capita household income (STVPI = 8.78%, 95% CI: 5.36%–13.73%), physical activity (8.62%, 95% CI: 5.64%–13.01%), distance to major roads (6.42%, 95% CI: 3.72%–10.50%), number of hospitalizations per capita (5.63%, 95% CI: 2.64%–11.03%), adequate sleep (5.21%, 95% CI: 2.26%–10.36%), and clean kitchen environment (4.42%, 95% CI: 1.33%–13.26%) were identified as the six key contributors (Table 2).

**Table 2:** Contributory factors to cognitive impairment

Indicators (Unit)	STVPI (%)	95%CI
Population demographics		
Family sizes	1.58	0.29–7.74
Per capita level of education (Year)	4.13	1.29–11.40
Annual household income per capita (RMB yuan)	8.78	5.36–13.73
Prevalence of multimorbidity among family members	2.29	0.40–9.31
Lifestyle		
Obesity	2.63	0.49–12.20
Adequate sleep	5.21	2.26–10.36
Physical activity	8.62	5.64–13.01
Healthy diet	2.08	0.34–9.90
Smoking (%)	3.20	0.91–9.32
Drinking (%)	1.45	0.25–6.95
blood pressure control rate(%)	2.15	0.20-10.13
Anxiety	3.13	1.05–7.61
Health care		
Annual health expenditure per capita (RMB yuan)	1.57	1.33–12.15
Disease duration	4.01	1.53–8.81
Number of hospitalizations per capita	5.63	2.64–11.03
WASH		
Access to clean cooking (%)	1.97	0.32–10.07
Access to clean kitchen environment	4.42	1.33–13.26
Access to safe water (%)	2.67	0.37–14.48
Access to sanitation facilities	1.65	0.25–9.27
Distance to major roads	6.42	3.72–10.50

MGWR model results: spatial heterogeneity. These six variables (STVPI > 5%) were subsequently incorporated into MGWR to examine whether their effects varied spatially across communities.

**Fig. 4:**
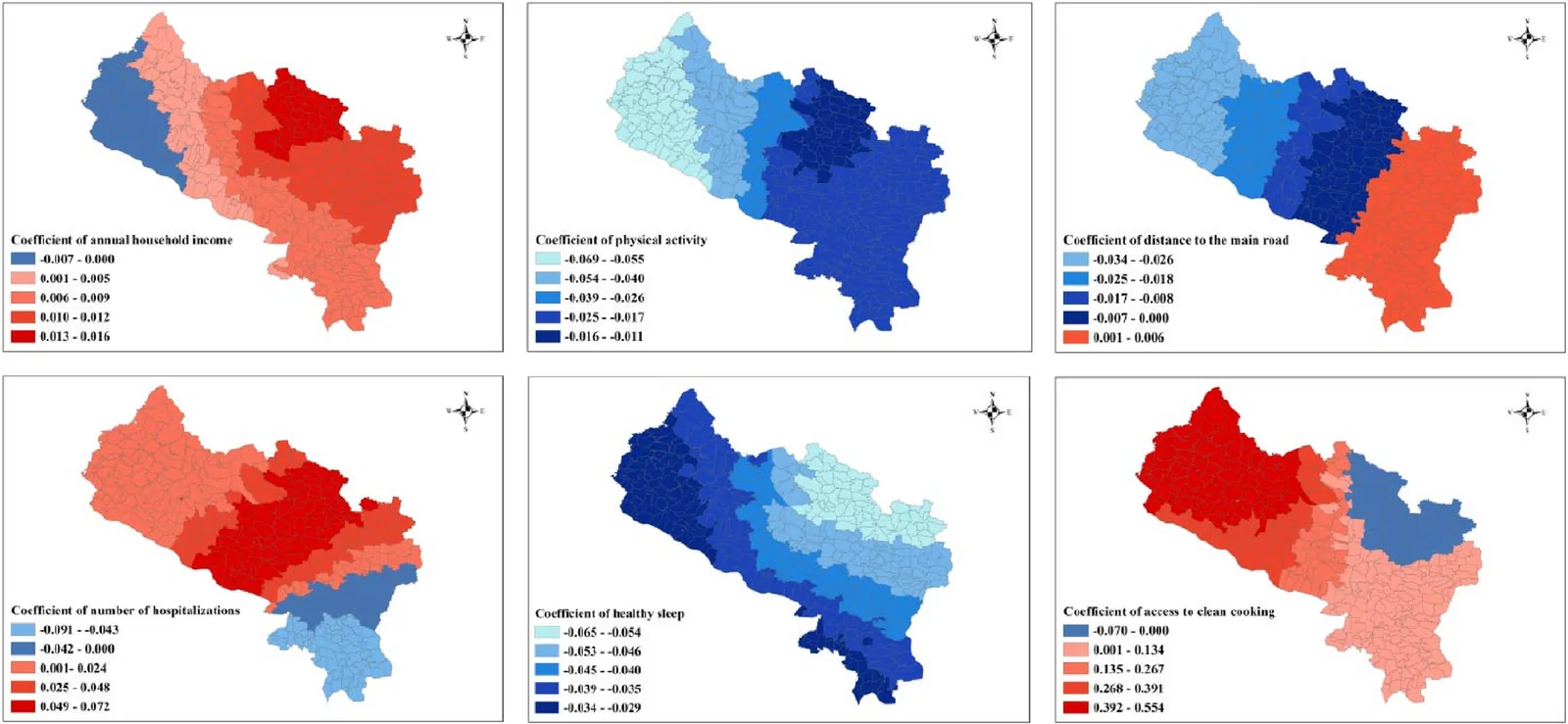
Spatial distribution of community-level MGWR regression coefficients for selected key influencing factors of cognitive impairment in Jia County. Per capita household income (coefficient range: −0.007 to 0.016). Physical activity (coefficient range: −0.069 to −0.011; spatially stable protective effect). Adequate sleep (coefficient range: −0.065 to −0.029; spatially stable protective effect). Distance to major roads (coefficient range: −0.034 to 0.006). Color scale ranges from dark blue (strongly negative/protective) to dark red (positive/risk-increasing)

MGWR results revealed distinct spatial patterns among the six key contributing factors, differentiating between spatially stable and spatially heterogeneous effects (Fig. 4). Physical activity and adequate sleep exhibited spatial stability, with consistently negative regression coefficients across all communities (physical activity: coefficient range = − 0.069 to − 0.011; adequate sleep: (coefficient range = − 0.065 to − 0.029), suggesting that their protective associations with cognitive function were uniform throughout Jia County, independent of community location. In contrast, the remaining four factors exhibited significant spatial heterogeneity in their regression coefficients. Per capita household income demonstrated a predominantly protective effect (coefficient range = − 0.007 to 0.016), with the strongest negative coefficients concentrated in north-central communities. Distance to major roads showed spatially varying associations (coefficient range = − 0.034 to 0.006), with the most pronounced coefficients in eastern communities. The number of hospitalizations per capita displayed the widest coefficient variation (coefficient range = − 0.091 to 0.072), with protective effects primarily observed in communities distant from the county center. Clean kitchen environment exhibited the most spatially concentrated risk-increasing coefficients (coefficient range = − 0.070 to 0.554), predominantly in western communities. These spatial patterns indicate that the associations between these factors and cognitive impairment vary substantially across locations, underscoring the insufficiency of uniform county-wide interventions.

## Discussion

### Prevalence and spatial distribution of cognitive impairment

Based on community survey data from 18,963 elderly hypertensive individuals in Jia County, a rural region of northern China, this study found that cognitive impairment exhibits significant heterogeneity at the village level. This heterogeneity manifested as high-prevalence clusters primarily concentrated in the contiguous northern and central regions. In these clusters, the prevalence of cognitive impairment exceeded the county average by more than 10% points and was accompanied by a high comorbidity burden, low health examination coverage, and poor living conditions, suggesting cumulative effects from multiple risk factors. A significant Moran’s I value confirmed that this spatial distribution pattern is non-random and exhibits significant positive spatial autocorrelation. This finding indicates that cognitive impairment is not uniformly distributed among rural elderly hypertensive populations but is intrinsically linked to specific geographical, social, and environmental contexts.

### Spatial clustering of cognitive impairment

This study revealed significant spatial heterogeneity in cognitive impairment prevalence, with distinct low-prevalence zones in urban and northwestern rural communities and high-prevalence zones in north-central rural communities. This village-level spatial heterogeneity within a single county adds important evidence to the literature, which has largely examined geographic disparities in cognitive health at national or provincial scales. Our findings echo studies on spatial clustering of other chronic conditions—such as diabetes and hypertension—which consistently demonstrate meaningful within-county variation across communities [27, 28]. The identification of high-high clustering in Anliang Town has direct implications for resource allocation. Priority should be given to establishing cognitive screening and early intervention networks in these identified high-burden areas [29]. GIS tools should be further leveraged to monitor spatial changes in cognitive impairment burden and guide deployment of community health workers to underserved high-risk areas [30].

### Spatial contributions and heterogeneity of influencing factors

Physical activity emerged as the single most important contributor to spatial variation in cognitive impairment (STVPI = 8.62%), with spatially stable protective effects across all communities. This finding is consistent with extensive evidence linking physical activity to reduced cognitive decline in older adults [31, 32]. The spatial stability of this association may reflect the predominantly agricultural character of Jia County, where elderly residents remain engaged in farming and physical labor, resulting in relatively uniform physical activity levels across communities—a pattern that contrasts with urban settings where neighborhood-level variation in activity is greater [7]. Adequate sleep similarly demonstrated a spatially stable protective effect (STVPI = 5.21%), consistent with established evidence that sleep disturbance accelerates cognitive decline in hypertensive older adults [33]. The uniform nature of this association suggests that sleep health promotion would benefit all communities equally.

The study further revealed that the influence of key factors on the prevalence of cognitive impairment among elderly hypertensive patients exhibits significant spatial heterogeneity. Specifically, annual household income, number of hospitalizations, and clean cooking conditions significantly impact cognitive impairment, with their effects varying considerably across geographic locations. Previous studies have primarily focused on establishing global associations between risk factors and cognitive impairment, often neglecting spatial non-stationarity in their conclusions and practical applications [34, 35]. For instance, while higher household income is theoretically protective against cognitive impairment, and increased hospitalizations may mitigate cognitive damage, interventions based solely on these global relationships—such as promoting higher income, increased hospitalization, and clean cooking—have yielded inconsistent results in practice [36, 37]. The spatial distribution of MGWR coefficients (Fig. 4) further reveals the geographic directionality of these heterogeneous effects. Per capita household income showed a protective effect concentrated in north-central communities—precisely the identified high-high clusters—suggesting that income poverty is a spatially localized driver of cognitive impairment burden in this region [29, 38]. Number of hospitalizations showed protective coefficients primarily in communities distant from the county center, reflecting inadequate healthcare access in peripheral rural areas [39]. Clean kitchen environment showed the strongest risk-increasing coefficients in western communities, consistent with evidence linking indoor air pollution to cognitive decline through neuroinflammatory pathways [40]. These spatially explicit patterns confirm that uniform county-wide policies are insufficient; effective prevention requires location-specific responses—income support in north-central communities, inpatient care quality improvement in peripheral areas, and clean cooking promotion in western communities.

### Advantages and limitations

This study possesses three key strengths: First, it leverages large-scale survey data from a complete rural population in northern China, which significantly enhances the stability and representativeness of the spatial modeling. Second, it accounts for both spatial variability and multi-scale effects, addressing major shortcoming of traditional GWR in terms of causal interpretation and scale resolution. Third, the innovative development of the STVPI indicator allows quantification of variable contributions across distinct geographic units, providing a robust evidence base for prioritizing interventions and designing resource-efficient regional strategies. Collectively, these strengths enhance the translational potential of the findings for public health policy.

Several limitations should be acknowledged. First, cognitive function was assessed using the MoCA, a validated screening tool; however, clinical diagnostic confirmation was not performed, and MoCA scores may be influenced by educational level, cultural factors, and examiner variability. Second, the cross-sectional design precludes causal inference; longitudinal studies are needed to establish temporal relationships between identified risk factors and cognitive decline. Third, the use of administrative villages as spatial units may introduce ecological bias and is subject to the MAUP; sensitivity analyses across alternative spatial scales would strengthen confidence in the observed patterns. Fourth, although MGWR accounts for multi-scale spatial heterogeneity, coefficient estimates may be sensitive to sample size and spatial configuration, and replication in other rural counties is needed to confirm generalizability.

## Conclusions

Cognitive impairment among rural elderly hypertensive patients in Jia County exhibits significant spatial clustering, with high-burden communities concentrated in the central-northern region characterized by cumulative socioeconomic and environmental disadvantage. Lifestyle factors—particularly physical activity and adequate sleep—are the primary modifiable determinants of spatial variation and demonstrate spatially uniform protective effects, making them suitable targets for county-wide behavioral intervention programs. In contrast, socioeconomic conditions, healthcare utilization, and living environment factors exhibit significant spatial heterogeneity, necessitating geographically differentiated responses: income support in the poorest north-central communities, inpatient care quality improvement in communities distant from the county center, and clean cooking energy promotion concentrated in western communities.

The integration of BSVC and MGWR models provides a replicable framework for identifying high-risk geographic areas and quantifying spatially heterogeneous risk contributions, supporting precision public health practice in resource-limited settings. Future research should validate these patterns using longitudinal designs and multi-county samples to strengthen causal inference and generalizability.

## Supplementary Information

Supplementary Material 1.

## Data Availability

The datasets used and/or analyzed during the current study are available from the corresponding author on reasonable request.

## Abbreviations

BSVC: Bayesian Spatial Variable Coefficient
MGWR: Multi-Scale Geographically Weighted Regression
MoCA: Montreal Cognitive Assessment
OLS: Ordinary least squares
STVPI: Spatial Target Variable Contribution Index
VIF: Variance inflation factor
CI: Confidence interval
SD: Standard deviation
WASH: Water, sanitation and hygiene
MAUP: Modifiable Areal Unit Problem
GAD-7: Generalized anxiety disorder 7-item scale
GWR: Geographically Weighted Regression
LISA: Local Indicators of Spatial Association
GSA: Global spatial autocorrelation
NBPHD: National Basic Public Health Database

## References

[1] Jia L, Du Y, Chu L, et al. Prevalence, risk factors, and management of dementia and mild cognitive impairment in adults aged 60 years or older in China: a cross-sectional study. Lancet Public Health. 2020;5(12):e661–71. 10.1016/s2468-2667(20)30185-7. 33271079 10.1016/S2468-2667(20)30185-7

[2] Petersen RC, Lopez O, Armstrong MJ, et al. Practice guideline update summary: Mild cognitive impairment [RETIRED]: Report of the Guideline Development, Dissemination, and Implementation Subcommittee of the American Academy of Neurology. Neurology. 2018;90(3):126–35. 10.1212/wnl.0000000000004826. 29282327 10.1212/WNL.0000000000004826 PMC5772157

[3] Ungvari Z, Toth P, Tarantini S, et al. Hypertension-induced cognitive impairment: from pathophysiology to public health. Nat Rev Nephrol. 2021;17(10):639–54. 10.1038/s41581-021-00430-6. 34127835 10.1038/s41581-021-00430-6 PMC8202227

[4] Zheng G, Zhou B, Fang Z, et al. Long-Term Visit-to-Visit Blood Pressure Variability and Cognitive Decline Among Patients With Hypertension: A Pooled Analysis of 3 National Prospective Cohorts. J Am Heart Assoc. 2024;13(13):e035504. 10.1161/jaha.124.035504. 38934858 10.1161/JAHA.124.035504 PMC11255695

[5] Guo Y, Liang R, Ren J, et al. Cognitive status and its risk factors in patients with hypertension and diabetes in a low-income rural area of China: A cross-sectional study. Int J Geriatr Psychiatry. 2023;38(10):e6010. 10.1002/gps.6010. 37794769 10.1002/gps.6010

[6] Guo D, Li T, Yang Q, et al. Relationship between Body Roundness Index and cognitive impairment in middle-aged and older adults: a population-based cross-sectional study. Front Aging Neurosci. 2025;17:1522989. 10.3389/fnagi.2025.1522989. 40046784 10.3389/fnagi.2025.1522989 PMC11879953

[7] Wei J, Miao Y, Zhang J, et al. Spatial heterogeneity of blood pressure control and its influencing factors in elderly patients with essential hypertension: A small-scale spatial analysis. Health Place. 2025;92:103428. 10.1016/j.healthplace.2025.103428. 39983658 10.1016/j.healthplace.2025.103428

[8] Ahmed A, Kheraj, Rocha J, et al. Asthma prevalence and risk factors in Poonch and Rajouri districts, India: an epidemiological and geospatial analysis. Sci Rep. 2025;15(1):23014. 10.1038/s41598-025-07482-9. 40595298 10.1038/s41598-025-07482-9 PMC12216878

[9] Zhang M, Shi Y, Zhou B, et al. Prevalence, awareness, treatment, and control of hypertension in China, 2004-18: findings from six rounds of a national survey. BMJ. 2023;380:e071952. 10.1136/bmj-2022-071952. 36631148 10.1136/bmj-2022-071952 PMC10498511

[10] Li Y, Wang L, Feng X, et al. Geographical variations in hypertension prevalence, awareness, treatment and control in China: findings from a nationwide and provincially representative survey. J Hypertens. 2018;36(1):178–87. 10.1097/hjh.0000000000001531. 29210864 10.1097/HJH.0000000000001531

[11] Liu Q, Wu S, Lei Y, et al. Exploring spatial characteristics of city-level CO(2) emissions in China and their influencing factors from global and local perspectives. Sci Total Environ. 2021;754:142206. 10.1016/j.scitotenv.2020.142206. 32920414 10.1016/j.scitotenv.2020.142206

[12] Wang L, BingganWei, Li Y, et al. A study of air pollutants influencing life expectancy and longevity from spatial perspective in China. Sci Total Environ. 2014;487:57–64. 10.1016/j.scitotenv.2014.03.142. 24768912 10.1016/j.scitotenv.2014.03.142

[13] He CYY, Zhou Z, Kan MMP, et al. Modifiable risk factors for mild cognitive impairment among cognitively normal community-dwelling older adults: A systematic review and meta-analysis. Ageing Res Rev. 2024;99:102350. 10.1016/j.arr.2024.102350. 38942197 10.1016/j.arr.2024.102350

[14] Deng Y, Zhao S, Cheng G, et al. The Prevalence of Mild Cognitive Impairment among Chinese People: A Meta-Analysis. Neuroepidemiology. 2021;55(2):79–91. 10.1159/000512597. 33756479 10.1159/000512597

[15] Kim H, Lee S, Ku BD, et al. Associated factors for cognitive impairment in the rural highly elderly. Brain Behav. 2019;9(5):e01203. 10.1002/brb3.1203. 30932371 10.1002/brb3.1203 PMC6520289

[16] Zhang H, Li S. Carbon emissions’ spatial-temporal heterogeneity and identification from rural energy consumption in China. J Environ Manage. 2022;304:114286. 10.1016/j.jenvman.2021.114286. 34915389 10.1016/j.jenvman.2021.114286

[17] Liu Y, Gao X, Zhang Y, et al. Geographical variation in dementia prevalence across China: a geospatial analysis. Lancet Reg Health West Pac. 2024;47:101117. 10.1016/j.lanwpc.2024.101117. 38974661 10.1016/j.lanwpc.2024.101117 PMC11225804

[18] Liu J, Liu W, Mi L, et al. Incidence and mortality of multiple myeloma in China, 2006–2016: an analysis of the Global Burden of Disease Study 2016. J Hematol Oncol. 2019;12(1):136. 10.1186/s13045-019-0807-5. 31823802 10.1186/s13045-019-0807-5 PMC6905074

[19] Ahmed A, Kheraj, Mohammadi A, et al. Understanding the factors associated with hypertension in the Western Himalaya: a GIS integrated analysis. BMC Public Health. 2025;25(1):3186. 10.1186/s12889-025-24469-3. 41029665 10.1186/s12889-025-24469-3 PMC12487095

[20] Yuan Y, Cave M, Xu H, et al. Exploration of spatially varying relationships between Pb and Al in urban soils of London at the regional scale using geographically weighted regression (GWR). J Hazard Mater. 2020;393:122377. 10.1016/j.jhazmat.2020.122377. 32114137 10.1016/j.jhazmat.2020.122377

[21] Anderson T, Herrera D, Mireku F, et al. Geographical Variation in Social Determinants of Female Breast Cancer Mortality Across US Counties. JAMA Netw Open. 2023;6(9):e2333618. 10.1001/jamanetworkopen.2023.33618. 37707814 10.1001/jamanetworkopen.2023.33618 PMC10502521

[22] Ogunsakin RE, Ginindza TG. Bayesian Spatial Modeling of Diabetes and Hypertension: Results from the South Africa General Household Survey. Int J Environ Res Public Health. 2022;19(15). 10.3390/ijerph19158886. 10.3390/ijerph19158886 PMC9331550 35897258

[23] Song C, Shi X, Bo Y, et al. Exploring spatiotemporal nonstationary effects of climate factors on hand, foot, and mouth disease using Bayesian Spatiotemporally Varying Coefficients (STVC) model in Sichuan, China. Sci Total Environ. 2019;648:550–60. 10.1016/j.scitotenv.2018.08.114. 30121533 10.1016/j.scitotenv.2018.08.114

[24] Nasreddine ZS, Phillips NA, Bédirian V, et al. The Montreal Cognitive Assessment, MoCA: a brief screening tool for mild cognitive impairment. J Am Geriatr Soc. 2005;53(4):695–9. 10.1111/j.1532-5415.2005.53221.x. 15817019 10.1111/j.1532-5415.2005.53221.x

[25] Ciesielska N, Sokołowski R, Mazur E, et al. Is the Montreal Cognitive Assessment (MoCA) test better suited than the Mini-Mental State Examination (MMSE) in mild cognitive impairment (MCI) detection among people aged over 60? Meta-analysis. Psychiatr Pol. 2016;50(5):1039–52. 10.12740/pp/45368. 27992895 10.12740/PP/45368

[26] Xia Y, Weller DE, Williams MN, et al. Using Bayesian hierarchical models to better understand nitrate sources and sinks in agricultural watersheds. Water Res. 2016;105:527–39. 10.1016/j.watres.2016.09.033. 27676387 10.1016/j.watres.2016.09.033

[27] Kauhl B, Schweikart J, Krafft T, et al. Do the risk factors for type 2 diabetes mellitus vary by location? A spatial analysis of health insurance claims in Northeastern Germany using kernel density estimation and geographically weighted regression. Int J Health Geogr. 2016;15(1):38. 10.1186/s12942-016-0068-2. 27809861 10.1186/s12942-016-0068-2 PMC5094025

[28] Yuan M, Kennedy KM. Utility of Environmental Complexity as a Predictor of Alzheimer’s Disease Diagnosis: A Big-Data Machine Learning Approach. J Prev Alzheimers Dis. 2023;10(2):223–35. 10.14283/jpad.2023.18. 36946449 10.14283/jpad.2023.18 PMC11416139

[29] Hunt JFV, Vogt NM, Jonaitis EM, et al. Association of Neighborhood Context, Cognitive Decline, and Cortical Change in an Unimpaired Cohort. Neurology. 2021;96(20):e2500. 10.1212/wnl.0000000000011918. 33853894 10.1212/WNL.0000000000011918 PMC8205478

[30] Kaneko Y, Takano T, Nakamura K. Visual localisation of community health needs to rational decision-making in public health services. Health Place. 2003;9(3):241–51. 10.1016/s1353-8292(02)00056-4. 12810331 10.1016/s1353-8292(02)00056-4

[31] Mohanty SK, Pedgaonkar SP, Upadhyay AK, et al. Awareness, treatment, and control of hypertension in adults aged 45 years and over and their spouses in India: A nationally representative cross-sectional study. PLoS Med. 2021;18(8):e1003740. 10.1371/journal.pmed.1003740. 34428221 10.1371/journal.pmed.1003740 PMC8425529

[32] Liu Q, Ni W, Zhang L, et al. Comparative efficacy of various exercise interventions on depression in older adults with mild cognitive impairment: A systematic review and network meta-analysis. Ageing Res Rev. 2023;91:102071. 10.1016/j.arr.2023.102071. 37704052 10.1016/j.arr.2023.102071

[33] Boa Sorte Silva NC, Barha CK, Erickson KI, et al. Physical exercise, cognition, and brain health in aging. Trends Neurosci. 2024;47(6):402–17. 10.1016/j.tins.2024.04.004. 38811309 10.1016/j.tins.2024.04.004

[34] Ozemek C, Tiwari S, Sabbahi A, et al. Impact of therapeutic lifestyle changes in resistant hypertension. Prog Cardiovasc Dis. 2020;63(1):4–9. 10.1016/j.pcad.2019.11.012. 31756356 10.1016/j.pcad.2019.11.012 PMC7257910

[35] Pickering TG. Lifestyle modification and blood pressure control: is the glass half full or half empty? JAMA. 2003;289(16):2131–2. 10.1001/jama.289.16.2131. 12709472 10.1001/jama.289.16.2131

[36] Wagner MK, Berg SK, Hassager C, et al. Cognitive impairment and psychopathology in sudden out-of-hospital cardiac arrest survivors: Results from the REVIVAL cohort study. Resuscitation. 2023;192:109984. 10.1016/j.resuscitation.2023.109984. 37797716 10.1016/j.resuscitation.2023.109984

[37] Shen C, Rolls ET, Cheng W, et al. Associations of Social Isolation and Loneliness With Later Dementia. Neurology. 2022;99(2):e164. 10.1212/wnl.0000000000200583. 35676089 10.1212/WNL.0000000000200583

[38] Chu L, Wu Y, Karjalainen H, et al. Lifespan exposures to rural-urban conditions and later-life cognitive function. Alzheimers Dement. 2025;21(6):e70267. 10.1002/alz.70267. 40538048 10.1002/alz.70267 PMC12179330

[39] Liu D, Li L, An L, et al. Urban-rural disparities in mild cognitive impairment and its functional subtypes among community-dwelling older residents in central China. Gen Psychiatr. 2021;34(5):e100564. 10.1136/gpsych-2021-100564. 34790888 10.1136/gpsych-2021-100564 PMC8557279

[40] Chan KH, Lam KBH, Kurmi OP, et al. Trans-generational changes and rural-urban inequality in household fuel use and cookstove ventilation in China: A multi-region study of 0.5 million adults. Int J Hyg Environ Health. 2017;220(8):1370–81. 10.1016/j.ijheh.2017.09.010. 28986011 10.1016/j.ijheh.2017.09.010 PMC6675611

