# Supplementary material for: Spatial heterogeneity and influencing factors of cognitive impairment among elderly hypertensive patients: evidence from rural communities in China
Source: BMC Geriatr. 2026 May 18;26:1069. doi: 10.1186/s12877-026-07587-4 (PMC13488023; doi:10.1186/s12877-026-07587-4)
Supplement: Supplementary file 1 [file 12877_2026_7587_MOESM1_ESM.docx]

Supplementary Materials

**Supplementary Figure 1** The study area and other comprehensive experimental areas.

**Supplementary Figure 2** Population Distribution of Survey Participants in Jia County.

**Supplementary Figure 3** Results of spatial autocorrelation analysis of cognitive impairment prevalence in elderly patients with hypertension.

**Supplementary Table 1** Comparison of related indicators of elderly patients with hypertension in Jia County and national statistics.

**Supplementary Table 2** Related indicators of elderly patients with hypertension in Jia County.

**Supplementary Figure 1** The study area and other comprehensive experimental areas.

**Supplementary Figure 2** Population Distribution of Survey Participants in Jia County

**Supplementary Figure 3** Results of spatial autocorrelation analysis of cognitive impairment prevalence in elderly patients with hypertension.

|  | Average level of Jia County | national average level |
| --- | --- | --- |
| Proportion of people aged over 65 years | 15.15% | 14.92% |
| Prevalence of hypertension among the elderly aged 65 years and over | 35.51% | 31.64% |

**Supplementary Table 1** Comparison of related indicators of elderly patients with hypertension in Jia County and national statistics

| Indicators | N（%） | **Cognitive impairment prevalence (%)** |
| --- | --- | --- |
| Total participants | 18963 (100) | 25.42 |
| Population demographics |  |  |
| Sex |  |  |
| Men | 8026（42.31） | 25.24 |
| Women | 10937（57.69） | 25.55 |
| Education years |  |  |
| 0 | 8490（44.77） | 23.07 |
| 1-6 | 6225（32.83） | 24.56 |
| 6-9 | 3128（16.50） | 32.93 |
| ≥9 | 1120（5.91） | 20.75 |
| Annual household income per  capita  <2400 | 4699（24.78） | 25.25 |
| 2400-4000 | 4681（24.68） | 27.57 |
| 4000-8600 | 4830（25.47） | 25.46 |
| >8600 | 4753（25.06） | 19.02 |
| **Lifestyle** |  |  |
| Obesity |  |  |
| Yes | 1744（9.20） | 23.55 |
| No | 17219（90.80） | 24.36 |
| Healthy sleep |  |  |
| Yes | 7997（42.17） | 23.50 |
| No | 10966（57.83） | 26.73 |
| Physical activity |  |  |
| Yes | 14709（77.57） | 22.35 |
| No | 4254（22.43） | 42.21 |
| Healthy diet |  |  |
| Yes | 6506（34.31） | 24.38 |
| No | 12457（65.69） | 25.31 |
| Smoking |  |  |
| Yes | 2134（11.25） | 22.54 |
| No | 16829（88.75） | 25.79 |
| Drinking |  |  |
| Yes | 2020（10.65） | 18.37 |
| No | 16943（89.35） | 26.31 |
| **Health care** |  |  |
| Annual health expenditure |  |  |
| ≤120 | 16115（84.98） | 23.15 |
| 120-480 | 2111（11.13） | 25.90 |
| 480-2400 | 564（2.97） | 25.21 |
| ≥2400 | 173（0.91） | 27.33 |
| Multimorbidity among family members |  |  |
| Yes | 3594（18.95） | 25.76 |
| No | 15369（81.05） | 24.34 |
| Anxiety |  |  |
| Yes | 5717（30.15） | 25.28 |
| No | 13246（69.85） | 25.02 |

**Supplementary Table 2** Related indicators of elderly patients with hypertension in Jia County
